# Effect of Waste Composite Plate Powders on the Mechanical, Durability and Microstructural Properties of Self-Compacting Mortars

**DOI:** 10.3390/ma19040810

**Published:** 2026-02-20

**Authors:** Yusuf Yıldırım, Alirıza İlker Akgönen, Serkan Etli

**Affiliations:** 1 Andırın Vocational School, Kahramanmaraş Sütçü İmam University, Kahramanmaraş 46410, Türkiye; 2 Department of Civil Engineering, Kahramanmaraş Sütçü İmam University, Kahramanmaraş 46100, Türkiye; ilkerakgonen@ksu.edu.tr (A.İ.A.); serkanetli@ksu.edu.tr (S.E.)

**Keywords:** self-compacting mortar, quartz-based composite plate, cultured marble, durability

## Abstract

This study investigates the effects of artificial plate powders with different compositions on the durability, physical, mechanical, and microstructural properties of self-compacting mortar (SCM). Waste quartz-based composite plate fragments and waste cultured marble pieces were ground into fine powders, and the resulting quartz-based plate powder (WQP) and cultured marble powder (WMP) were used as filler materials to partially replace cement at replacement levels of 0%, 5%, 10%, 15%, 20%, and 25% by mass. The workability of fresh mortars was evaluated using the mini slump flow test in accordance with EFNARC guidelines, while hardened specimens were tested for porosity, capillary water absorption, abrasion resistance, flexural strength, and compressive strength. In addition, specimens with a 25% replacement ratio that were exposed to temperatures of 300 °C, 600 °C, and 900 °C underwent mechanical testing, and their microstructures were analyzed using SEM and XRD. The results indicated that increasing replacement ratios generally reduced workability and mechanical strength, while increasing porosity and water absorption. However, low replacement levels slightly enhanced flexural strength due to the filler effect. SEM and XRD analyses revealed that the quartz in WQP maintained high thermal stability, and mortars containing WQP exhibited a denser, more coherent, and more homogeneous microstructure. Overall, the findings demonstrate that waste-based plate powders can serve as sustainable fillers in SCM, offering environmental benefits while maintaining acceptable mechanical and microstructural performance.

## 1. Introduction

Cement is considered one of the most important construction materials worldwide. The cement production process accounts for approximately 5–8% of global carbon dioxide (CO_2_) emissions [1]. During production, gaseous particulates are released into the environment, which can reduce visibility, deteriorate air quality, and contaminate water resources. Workers in cement plants and nearby residents may be exposed to various health risks, particularly respiratory diseases. They can be exposed to nitrogen oxides (NO_x_), sulfur dioxide (SO_2_), carbon monoxide (CO), volatile organic compounds, ammonia, chlorine, and hydrogen chloride [2]. High temperatures are required in rotary kilns for chemical reactions to occur, which are achieved through the consumption of substantial amounts of fossil fuels. In addition, processes such as raw material preparation and clinker grinding also consume significant amounts of energy [3].

With the rapid urbanization process, increasing construction activities have led to a significant rise in cement production. However, cement production is associated with several adverse impacts, including high energy consumption, greenhouse gas emissions, and environmental pollution [4]. In order to mitigate these environmental effects, research on alternative binder materials that can partially or completely replace cement has gained considerable momentum in recent years. In this context, geopolymers have emerged as a potential alternative to conventional cement due to their high compressive strength and superior durability [5].

Various industrial by-products and wastes have been investigated as partial replacements for cement or aggregates in concrete and mortar production. Commonly used materials include fly ash, silica fume, ground granulated blast furnace slag, and natural stone powders. Natural stones such as marble, granite, and basalt are often processed into fine powders and used as fillers in mortars or concrete; this has been shown to improve particle packing and contribute to sustainable material use [6]. In addition, the literature reports studies in which specific materials, such as used foundry sand, have been employed as fine aggregate substitutes [7].

Irmak Er & Yazıcıoğlu [8] investigated the feasibility of using granite waste sludge and sepiolite as partial cement replacement materials in self-compacting mortars. In the mortar mixes prepared with different replacement ratios, the fresh-state workability was evaluated, while the hardened mortars were comprehensively assessed in terms of mechanical strength, density, porosity, and water absorption. The findings indicate that the use of granite waste sludge and sepiolite at certain proportions can enhance the mechanical and durability properties of hardened mortars without adversely affecting the performance of fresh mortars. These results suggest that industrial by-products such as granite and sepiolite can be employed as sustainable and technically viable filler materials.

Benjeddou & Alwetaishi [9] investigated the feasibility of using waste sludge from the marble processing industry, processed into fine powder, as a mineral filler material. The authors reported that the powder consists primarily of calcium carbonate, exhibits inert behavior, and does not participate in cement hydration. Physical and chemical characterization tests indicated that the powder is very fine and well-graded, while hydraulic tests and cement–powder blend experiments demonstrated that mechanical strength was not adversely affected. Consequently, marble sludge powder can be safely utilized in concrete and mortars as a partial cement replacement or filler, offering a sustainable and environmentally friendly material option.

Hameed et al. [10] investigated the feasibility of using waste marble powder as an inert filler in self-compacting concrete. Mixes containing various dosages of marble powder were compared with a control mix without marble powder, and the fresh-state properties, including flow, viscosity, and passing ability, as well as the hardened-state properties, including compressive and flexural strengths, were evaluated. The study demonstrated that marble powder, up to certain dosages, maintained the fresh concrete performance and did not significantly compromise mechanical properties, indicating its potential as a sustainable inert filler material.

Karakurt & Dumangöz [11] comprehensively investigated the effects of using marble powder and blast furnace slag as filler materials on the fresh and hardened properties of self-compacting concrete. The study holistically evaluated the rheological behavior and durability characteristics, revealing that the incorporation of approximately 10% marble powder and 30% blast furnace slag preserved the workability of fresh concrete while improving mechanical and durability properties. The findings indicate that these two waste materials can be considered sustainable and technically viable filler materials.

Prakash et al. [12] examined the use of waste marble powder in cement-based composites. They replaced cement with marble powder at replacement levels of 0%, 5%, 10%, 20%, 30%, 40%, and 50%. The researchers analyzed water absorption, porosity, water permeability, compressive strength, splitting tensile strength, flexural strength, and microstructural properties. Their findings showed a slight increase in compressive strength in specimens containing up to 10% marble powder replacement.

Abouelnour et al. [13] studied the individual and combined use of marble powder, granite powder, and nano-alumina powder as partial replacements for Portland cement. Marble and granite powders were used at replacement levels of 2%, 4%, 6%, 8%, and 10% by weight of cement, while nano-alumina powder was used at 0.25%, 0.5%, 0.75%, 1%, and 1.25%. The study evaluated the physical properties, workability, mechanical performance, and microstructural characteristics of the mixtures. The highest splitting tensile, flexural, and compressive strength values were obtained at 6% replacement for marble and granite powders and at 1% for nano-alumina powder.

Sevinç & Durgun [14] produced mortar mixtures by partially replacing cement with barite, pumice, and basalt powders at replacement levels of 7.5% and 15%. The mortar specimens were subjected to elevated temperatures of 100, 200, 300, 400, 500, and 600 °C after being cured for 90 days. Two different cooling methods—air and water—were applied following the heating process. Before heating, the unit weight, ultrasonic pulse velocity, and compressive strength of the specimens were measured. After exposure to high temperatures, compressive strength, ultrasonic pulse velocity, and mass loss were determined. Additionally, TGA, SEM, and XRD analyses were performed. The results indicated that the specimens containing basalt were less affected by high temperatures compared to the others.

Huseien et al. [15] investigated the use of epoxy resin polymer as a self-healing agent in Portland cement-based mortars. The mortars were prepared with different epoxy resin contents (5–20% by weight of cement), and the effects on mechanical performance and microstructure were evaluated. The experimental results showed that 10% epoxy resin addition significantly improved compressive, flexural, and tensile strengths, while also reducing porosity and water absorption. Higher resin contents led to stabilization of the mechanical properties. This study shows that the interaction of epoxy resin with cement hydration, coupled with the self-healing mechanism, improves both mechanical and durability performance of cementitious mortars, adding to the knowledge on polymer-modified cementitious systems.

Abuqasim et al. [16] investigated the use of porcelain polishing residue (PPR), a fine powder generated during the surface polishing of porcelain tiles, as a partial cement replacement in Portland cement-based mortars. Mortars were prepared with different PPR contents (5–20% by weight of cement) and evaluated in terms of fresh properties, mechanical performance, and microstructure. The results showed that moderate PPR addition improved compressive, flexural, and tensile strengths, reduced porosity, and decreased water absorption, while higher replacement ratios stabilized the mechanical properties. The study demonstrated that PPR interacts with cement hydration to enhance the microstructure and durability of mortars, highlighting its potential as a sustainable, artificial plate filler in cementitious systems.

Bayraktar et al. [17] investigated the mechanical and durability properties of sustainable cement-based foam concretes incorporating waste ceramic powder (CP) and waste polyester fibers. In the study, ceramic powder was used as a partial cement replacement at 0%, 10%, and 20%, while polyester fibers were added to the mixtures at volume fractions ranging from 0 to 0.6%. The experimental results showed that the use of 10–20% ceramic powder improved the pore structure through a micro-filler effect and positively influenced durability-related properties such as water absorption, whereas the polyester fibers contributed to crack control and enhanced ductility. The study demonstrated that the performance of cementitious systems can be optimized through the appropriate use of ceramic-based waste powders and polymer additives.

Nowadays, as an alternative to plates made from natural stones such as marble and granite, artificial plates are manufactured using various chemical- and mineral-based components. These materials are widely employed in kitchen and bathroom countertops, worktops, as well as wall and ceiling claddings. Due to their smooth surface texture, wide range of colors and patterns, antibacterial properties, high mechanical strength, and inconspicuous joints, the use of artificial plates has been steadily increasing in recent years [18].

Artificial plates, commonly referred to as engineered stone materials, differ fundamentally from natural stones in both composition and production process. While natural marble and granite consist solely of mineral phases, engineered stone plates are manufactured by binding mineral aggregates with polymeric resins. This resin-bonded composite structure results in distinct physical, mechanical, and thermal behaviors compared to natural stone materials. In particular, the presence of organic binder phases influences particle characteristics, interaction with cementitious matrices, and degradation mechanisms under elevated temperatures. Therefore, artificial plate waste powders should be evaluated separately from natural stone powders when considering their use as filler materials in cement-based composites.

Similar to natural stone plates, the processing of artificial plates also generates production waste that cannot be reused. These wastes are mostly disposed of in landfill sites alongside other stone plate residues. However, the environmental impact of these artificial plate wastes, which are produced entirely from chemical components and resins, is considerably higher compared to that of natural stone waste.

Therefore, in order to both reduce environmental pollution and recover economic value from these wastes, it is of great importance to incorporate artificial plate waste into production processes through reuse or recycling methods. This approach aligns with sustainable production goals and contributes to increasing material efficiency in the construction sector. Based on the reviewed studies, Table 1 presents a comparative overview of the use of natural and engineered stone powders as cement replacement materials in SCM and SCC.

However, despite extensive studies on natural stone powders, limited research has focused on resin-containing artificial plate wastes, particularly regarding their behavior in self-compacting mortars under elevated temperatures. Therefore, this study aims to evaluate the feasibility of using artificial plate waste powders as sustainable filler materials in self-compacting mortars. Waste cultured marble (WMP) and quartz-based composite plate powders (WQP) were used as partial cement replacements at various replacement levels, and their effects on fresh properties, mechanical performance, durability, and microstructural characteristics were systematically investigated. In addition, selected specimens were exposed to elevated temperatures of 300 °C, 600 °C, and 900 °C to assess the thermal stability and microstructural evolution of mortars containing resin-bonded artificial plate powders.

## 2. Materials and Methods

### 2.1. Materials

The cement used in the study was CEM I 42.5 R Portland cement, produced by the KÇS Cement Factory, Kahramanmaraş, Turkey. The chemical, physical, and mechanical properties of the cement are summarized in Table 2.

**Figure 1:**
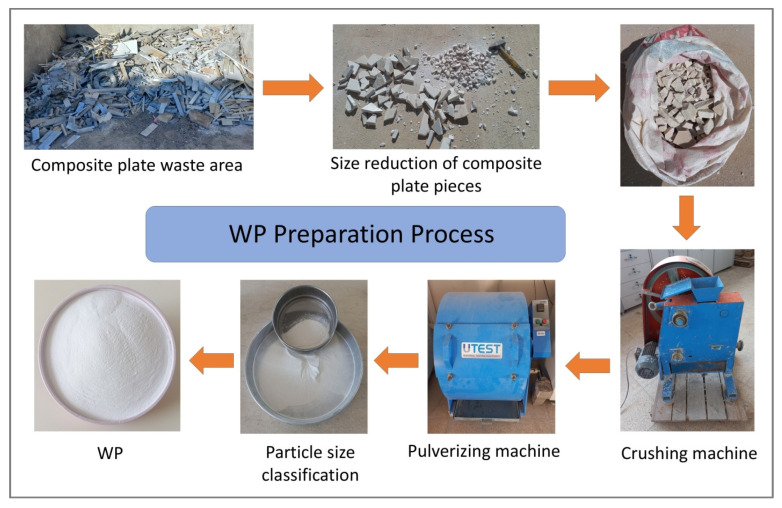
Flowchart of WP preparation process.

The waste powder (WP) materials used in this study were obtained from cultured marble and quartz-based composite plates collected from a marble waste site. Powders passing through a 0.063 mm sieve were selected for use. The particle densities of WMP and WQP were determined as 1.88 g/cm^3^ and 1.71 g/cm^3^, respectively. The chemical compositions of WMP and WQP are presented in Table 3. The experimental workflow and preparation procedure are illustrated in Figure 1.

**Figure 2:**
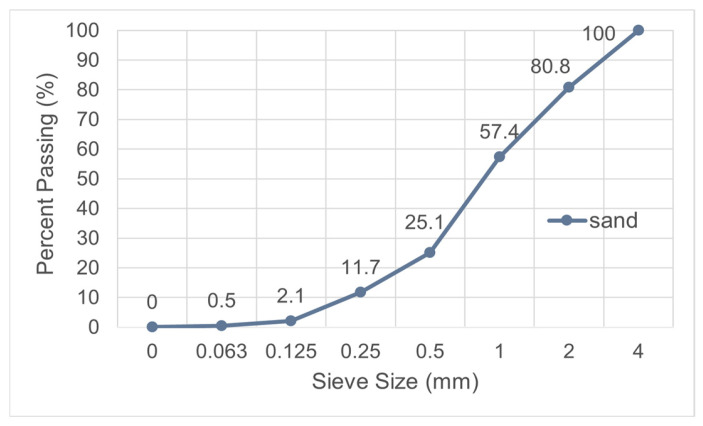
Granulometric curve of the sand.

Fine washed sand was sourced from the Narlı region of Kahramanmaraş and sieved through a 4 mm sieve to remove coarse particles. The granulometric curve of the sand is shown in Figure 2.

For the production of self-compacting mortars, a polycarboxylate ether-based superplasticizer conforming to the TS EN 934-2 standard was used [19]. The selected high-range water reducer (HRWR) was Sika Viscocrete ACE 450 (Istanbul, Türkiye), with a density ranging between 1.069 and 1.109 kg/L [20].

#### Mix Design

A total of 11 different mortar mixes, including the control mix, were prepared in this study. The replacement ratios of waste powder were set at 0%, 5%, 10%, 15%, 20%, and 25% by mass. The total binder (cement + waste powder) content was kept constant at 600 kg/m^3^. Different dosages of HRWR were used to achieve the desired workability. Although HRWR adjustments may have secondary and limited effects on mechanical and durability properties, the observed trends in compressive strength, flexural strength, porosity, and sorptivity are considered to be primarily influenced by the waste powder replacement levels. All mixes were prepared using a concrete mixer with a capacity of 56 dm^3^.

The specimens incorporating waste cultured marble powder were designated as MP, while those containing waste quartz-based composite plate powder were denoted as QP. The control specimens were labeled as REF. The numeral following each code indicates the mass percentage of waste powder used as a partial replacement for cement.

The mortars were produced and cast according to EFNARC guidelines. After 24 h, the specimens were demolded and placed in a curing pool. On the 7th day, three prism specimens from each set were removed from the curing pool and subjected to flexural and compressive strength tests. On the 28th day, all specimens were taken out of the curing pool and stored in laboratory conditions until testing.

**Figure 3:**
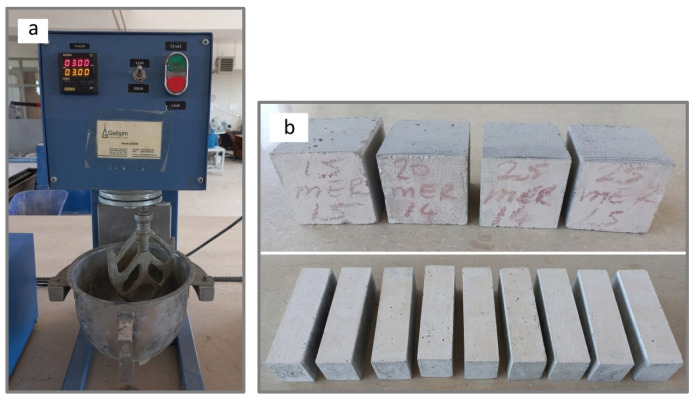
(**a**) Mixing constituents of SCMs. (**b**) Cubic and prismatic specimens.

At 28 days, tests including flexural strength, compressive strength, porosity, specific gravity, capillary water absorption, abrasion resistance, mechanical tests after exposure to elevated temperatures, and microstructural analyses were performed. Additionally, on the 56th day, flexural and compressive strengths of three prism specimens were determined. Figure 3 illustrates (a) the mixing of fresh mortar and (b) the hardened mortar specimens. Table 4 presents the mix quantities of cement mortars containing WMP and WQP.

### 2.2. Methodology

In this study, a total of 11 sets of specimens were prepared, including one set for the control group (REF) and five sets each for the MP and QP groups. Each set consisted of 21 prism specimens measuring 40 × 40 × 160 mm and 12 cube specimens measuring 71 × 71 × 71 mm. Among the prism specimens, 9 were designated for flexural and compressive strength tests, 3 for capillary water absorption tests, and 9 for flexural and compressive tests following exposure to elevated temperatures. Among the cube specimens, 3 were subjected to abrasion tests at ambient temperature (AT), while 9 were subjected to abrasion tests after high-temperature exposure. In total, 231 prism and 132 cube specimens were produced. For each mechanical and durability test, at least three specimens were tested, and the reported values represent the average of these measurements.

#### 2.2.1. Fresh State Testing Procedure

**Figure 4:**
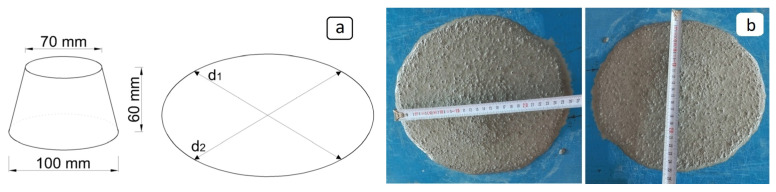
(**a**) Mini slump-flow test setup. (**b**) Mini-slump flow diameter.

To determine the flow diameter of the fresh mortar, a truncated cone-shaped mold was used, with a bottom diameter of 100 mm, a top diameter of 70 mm, and a height of 60 mm. The mold was filled with fresh mortar and then carefully lifted vertically to allow the mortar to spread freely. The diameters of the spread mortar were measured in two perpendicular directions, and the average of these two values was recorded as the flow diameter (Figure 4).

To determine the unit weight of the fresh mortar, a container with a known volume was filled with the fresh mix and weighed. The weight of the filled container was then divided by its volume to calculate the unit weight of the mortar.

#### 2.2.2. Exposure to Elevated Temperature

On the 28th day, 9 prism and 9 cube specimens from each set were removed from the curing pool and oven-dried at 105 ± 2 °C until they reached a constant mass. After drying, the specimens were weighed and placed in a high-temperature furnace.

**Figure 5:**
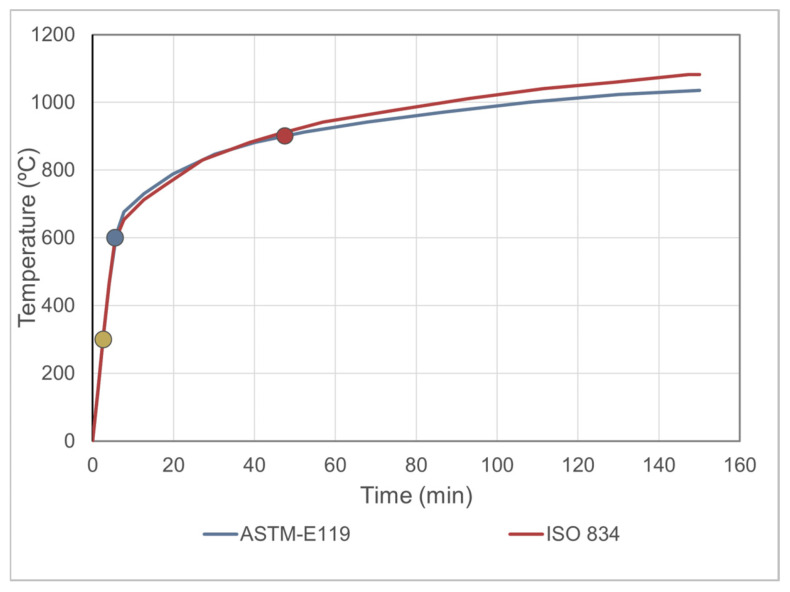
Standard time–temperature curves [20,21]. Experimental points are indicated as yellow for 300 °C, blue for 600 °C, and red for 900 °C.

The heating rate was adjusted according to the temperature-time curve specified in ISO 834 and ASTM E119 standards [21,22]. The heating curve followed is presented in Figure 5.

From each set,

3 prism and 3 cube specimens were heated to 300 °C,

3 prism and 3 cube specimens to 600 °C,

3 prism and 3 cube specimens to 900 °C.

**Figure 6:**
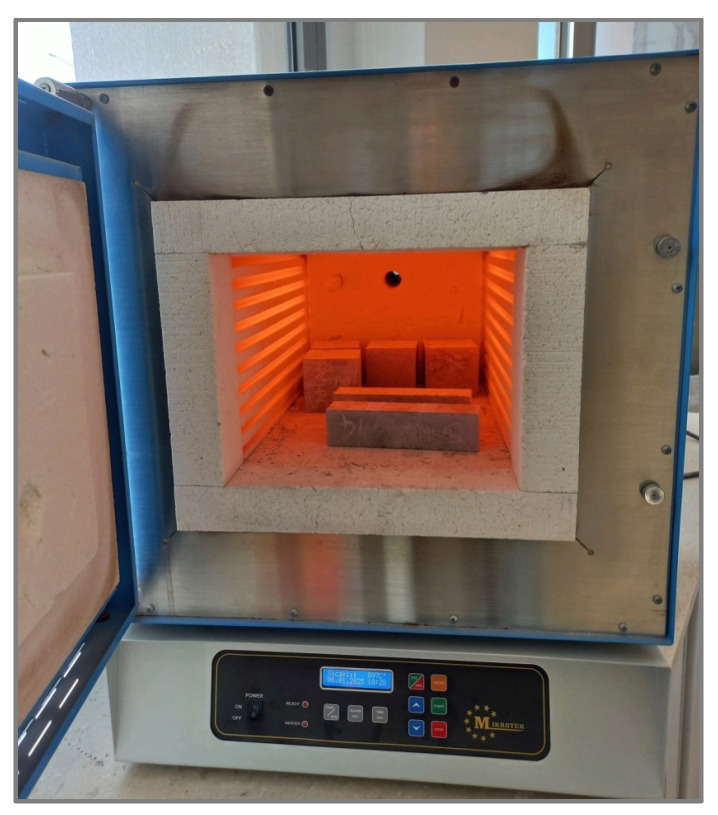
High-temperature furnace.

Once the target temperatures were reached, the specimens were held at the respective temperatures for 30 min inside the furnace (Figure 6). After the heating period, all specimens were removed and allowed to cool naturally in ambient air. Since the natural cooling regime can influence microstructural changes and mechanical properties, this is acknowledged as a limitation of the study.

Following the cooling process, flexural and compressive strength tests were conducted on the prism specimens, while abrasion resistance tests were carried out on the cube specimens.

#### 2.2.3. Flexural and Compressive Strength Test

The 40 × 40 × 160 mm prism specimens were subjected to flexural strength tests on the 7th, 28th, and 56th days in accordance with the TS EN 196-1 standard [23].

**Figure 7:**
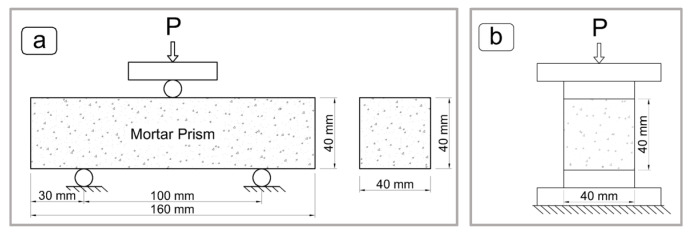
Schematic diagrams of mortar tests according to TS EN 196-1: (**a**) flexural test, (**b**) compressive test.

Following the flexural test, each of the two broken halves of the prism specimens was used to perform compressive strength tests, also in accordance with TS EN 196-1 [23]. Schematic diagrams of the flexural and compressive strength tests are presented in Figure 7.

The flexural strength ($f_{flex}$) was calculated using the following equation:

(1) $$f_{flex}=\frac{3Pl}{2bh^{2}}\;,$$

where $f_{flex}$ flexural strength (MPa), P maximum load at failure (N), l span between supports (mm), b specimen width (mm), h specimen height (mm).

The compressive strength ($f_{c}$) was determined using the following formula:

(2) $$f_{c}=\frac{P}{A}\;,$$

where $f_{c}$ compressive strength (MPa), P maximum load at failure (N), A cross-sectional area of the specimen (mm^2^).

#### 2.2.4. Sorptivity Test

The sorptivity test is a method used to determine the amount of water that penetrates through capillary pores on the surface of mortar or concrete [24]. In this study, sorptivity tests were conducted on 28-day cured prism specimens in accordance with ASTM C1585–13 [25].

All lateral surfaces of the prism specimens, except the bottom surface in contact with water, were sealed using a waterproof coating material. After sealing, the specimens were weighed and placed into shallow water containers so that only the bottom surface was submerged.

**Figure 8:**
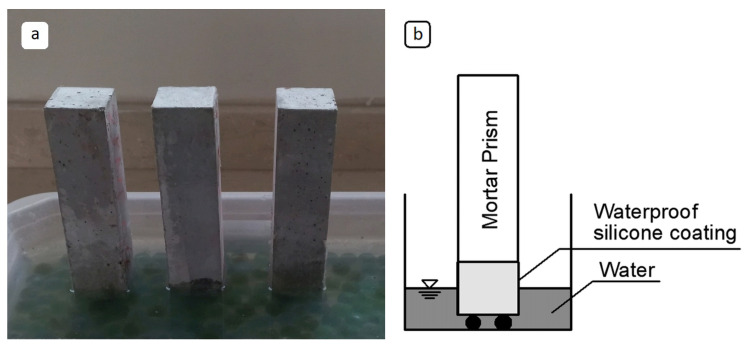
(**a**) Sorptivity test set-up. (**b**) Schematic illustration of the sorptivity test set-up.

At 5, 10, 30, 60, 240, and 1440 min, the specimens were removed from the water, gently wiped with a towel to remove surface moisture, and then weighed again. The sorptivity test set-up is shown in Figure 8, including (a) the experimental arrangement with specimens submerged in water and (b) a schematic illustration of the setup.

#### 2.2.5. Porosity and Bulk Specific Gravity Tests

After the completion of the curing process, the mortar specimens were carefully removed from the curing tank on the 28th day to determine their physical properties. Three fundamental weight measurements were conducted to evaluate the porosity and specific gravity of the mortar specimens.

Firstly, the suspended weights of the specimens in water ($M_{w}$) were measured using an Archimedes balance, which operates based on the Archimedes principle. This measurement corresponds to the volume of water displaced by the specimen and therefore serves as a primary parameter in calculating the total volume of the specimen.

Subsequently, the specimens were taken out of the water, and the excess surface water was gently removed using a soft towel to prevent any influence on subsequent measurements. The mass obtained after this procedure was recorded as the saturated surface-dry weight ($M_{ssd}$). This condition represents the equilibrium state in which all pores are completely filled with water while no free water remains on the specimen’s surface.

In the final stage, the specimens were oven-dried at 105 ± 2 °C until a constant mass was achieved, ensuring that all free water within the specimen was completely removed. Upon reaching a constant weight, the oven-dry mass ($M_{d}$) of each specimen was accurately measured.

Using these three weight measurements—suspended weight in water ($M_{w}$), saturated surface-dry weight ($M_{ssd}$), and oven-dry weight ($M_{d}$)—the porosity (n) and bulk specific gravity ($G_{bulk}$) of the mortar specimens were calculated according to the following equations.

(3) $$n=\frac{M_{ssd}-M_{d}}{M_{ssd}-M_{w}}\times 100\;,$$

(4) $$G_{bulk}=\frac{M_{d}}{M_{ssd}-M_{w}}\;,$$

where n total porosity (%), $G_{bulk}$ bulk specific gravity (dimensionless), $M_{ssd}$ saturated surface-dry weight (g), $M_{d}$ oven-dry weight (g), $M_{w}$ suspended weight in water (g).

#### 2.2.6. Abrasion (Loss) Resistance Tests

Abrasion resistance testing was carried out using the Böhme abrasion testing device. This test is particularly applied to evaluate the surface strength and service life of cement-based materials, such as mortar and concrete.

For the test, cube-shaped specimens were placed in the designated chamber of the device. All four surfaces of each specimen were uniformly abraded by the rotational movement of the abrasive disc. During this process, a controlled amount of material was worn away from the surface, allowing the abrasion resistance of the material to be observed.

Upon completion of the test, the specimens were weighed using a precision balance. The difference between their initial and final masses was divided by the initial mass to calculate the mass loss, and these values were used to quantitatively assess the material’s resistance to abrasion.

#### 2.2.7. Microstructure Analysis

After a 28-day curing period, specimens containing 25% WP were exposed to temperatures of 300 °C, 600 °C, and 900 °C. Representative portions taken from these specimens were subjected to Scanning Electron Microscopy (SEM) and X-ray Diffraction (XRD) analyses in order to characterize their microstructural and mineralogical properties.

SEM investigations were carried out using a Zeiss Evo LS 10 scanning electron microscope at Kahramanmaraş Sütçü İmam University, USKIM (University-Industry-Public Cooperation Development Application and Research Center), Kahramanmaraş, Turkey. Prior to imaging, the specimen surfaces were coated with a thin layer of gold to obtain high-resolution and well-defined images. Micrographs were acquired at magnifications ranging from 5000× to 10,000×, and all images are presented with a 1 µm scale bar.

Portions obtained from the same specimens were also analyzed by XRD to determine the phase composition and changes in crystalline structure. XRD measurements were performed using a Rigaku Miniflex 600 diffractometer at Munzur University, Rare Earth Elements Application and Research Center, Tunceli, Turkey. By jointly evaluating the results of SEM and XRD analyses, features such as matrix degradation, porosity, and changes in gel structure were assessed.

## 3. Results

### 3.1. Fresh Properties Results

**Figure 9:**
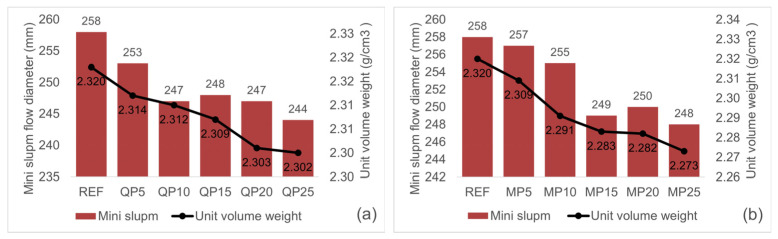
Mini slump and unit volume weight of fresh mortar for QP (**a**) and MP (**b**) specimens.

The EFNARC committee recommends that the mini slump flow of self-compacting mortars should fall within the range of 240–260 mm. Since the water content was maintained constant across all mixtures, achieving flow diameters within the specified range was accomplished through the use of varying dosages of high-performance water-reducing admixture (HRWR). As shown in Table 2, the required HRWR dosage increased with higher replacement ratios. The flow diameters of the fresh mortars are presented in Figure 9. Based on the data in Table 2 and Figure 9, it can be concluded that as the replacement ratio increases, the water demand rises and the workability of the mortars decreases.

The unit weight of mortars decreased with increasing replacement ratios, primarily due to the reduction in high-density cement content. Cement particles possess significantly higher density compared to the replacement materials, and their binding properties help reduce porosity in the matrix. Substituting cement with non-binding materials increases void content, making the observed decrease in unit weight an expected outcome.

Interestingly, mortars prepared with WQP, despite its lower particle density, exhibited higher unit weight than those prepared with WMP. This can be attributed to differences in particle morphology and surface characteristics. WQP particles are smoother and more hydrophobic, which limits water distribution, reduces air entrapment, and promotes denser packing. In contrast, WMP is calcite-based with rougher surfaces, favoring air entrapment and lower unit weight.

### 3.2. Porosity and Bulk Specific Gravity Test Results

Porosity is a critical parameter that directly influences the durability, permeability, and overall performance of mortar. In general, lower porosity indicates a denser microstructure and consequently improved mechanical properties and durability.

**Figure 10:**
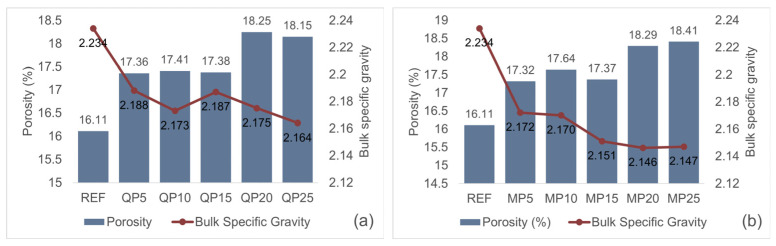
Porosity and bulk specific gravity of QP (**a**) and MP (**b**) specimens.

As the replacement ratio increases, the porosity of the mortars increases, while their bulk specific gravity decreases. This trend is primarily attributed to the substitution of high-density cement particles with lower-density powders, which reduces the total amount of solid material per unit volume. Additionally, the increase in replacement ratio leads to a higher number of voids within the mortar. The particle shape and surface roughness of the powders affect the distribution of water within the matrix, thereby contributing to the observed trends in porosity and bulk specific gravity. The porosity and bulk specific gravity results of QP and MP specimens are presented in Figure 10.

### 3.3. Sorptivity Test Results

Sorptivity is a crucial durability parameter that characterizes the rate at which a material absorbs water through capillary suction. Due to their superior workability, self-compacting mortars have the potential to develop a denser and more homogeneous microstructure compared to conventional mortars. This theoretically results in lower porosity and permeability. However, any degradation in the microstructure or an increase in the capillary pore system can adversely affect the expected superior durability performance of self-compacting mortars.

**Figure 11:**
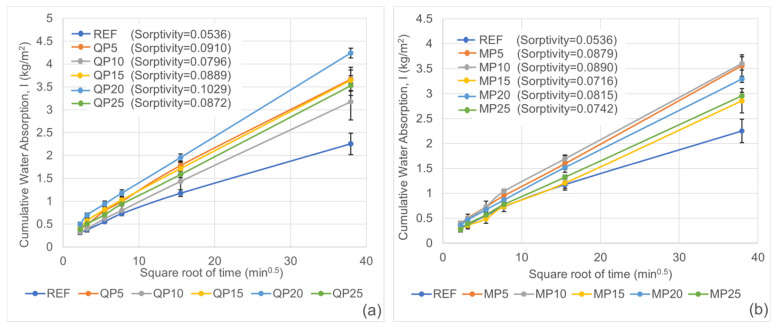
Sorptivity test results for QP (**a**) and MP (**b**) specimens.

The sorptivity test results for 28-day cured prism mortar specimens are presented in Figure 11. Among all groups, REF exhibited the lowest mass/area ratio, indicating the lowest capillary water absorption rate and therefore the highest resistance to external water ingress. As the WP replacement ratio increased, the capillary water absorption curves deviated further from that of the control mix, reaching higher mass/area ratios. This indicates that the incorporation of WP significantly increases both the capacity and rate of capillary water absorption in the mortars.

### 3.4. Flexural Strength Test Results

**Figure 12:**
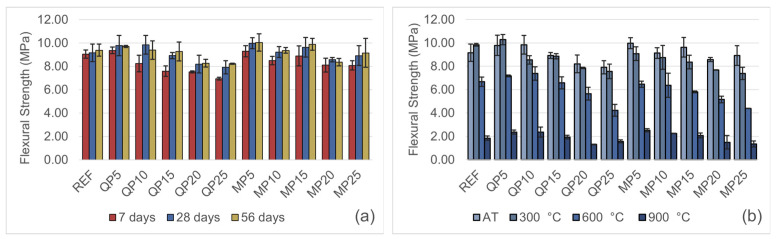
(**a**) Change in flexural strength at 7, 28, and 56 days of curing. (**b**) Flexural strength test results after exposure to AT, 300, 600, and 900 °C.

Figure 12a presents the flexural strength results at 7, 28, and 56 days. Both WQP and WMP exhibit favorable performance at low replacement levels in terms of flexural strength. However, higher replacement ratios generally lead to a reduction in flexural strength, with this decrease being more pronounced in the QP series specimens. At low replacement levels, the waste powders enhance particle packing density by filling the fine pores within the mortar matrix through their micro-filling effect. This reduces the void volume between cement particles, resulting in a more compact microstructure and more efficient stress transfer. The powders may also impart slight flexibility to the matrix, reducing brittleness and contributing to the observed strength increase.

At higher replacement ratios, however, the reduction in cement content leads to a decrease in binding capacity, a reduction in the quantity of cement hydration products, and the formation of a greater number of voids within the mortar structure. These factors collectively contribute to the observed decline in flexural strength.

The results clearly indicate that there exists an optimal replacement range for both waste materials, beyond which flexural strength begins to decrease. This optimal level is approximately 10% for WQP and may reach up to 15% for WMP.

The temperature-dependent flexural strength results are presented in Figure 12b. For the REF and QP5 specimens, a slight increase in flexural strength was observed at 300 °C compared to ambient temperature. This enhancement can be attributed to the partial release of bound water at lower temperatures, which may promote the partial closure of micro-voids and result in a denser matrix. Similar trends have been reported in the literature, where certain modified mortars demonstrated increased flexural strength around 300 °C due to filler effects and improved matrix integrity [26]. Nevertheless, for both specimen groups, an overall reduction in flexural strength was noted with increasing replacement ratio and exposure temperature.

**Figure 13:**
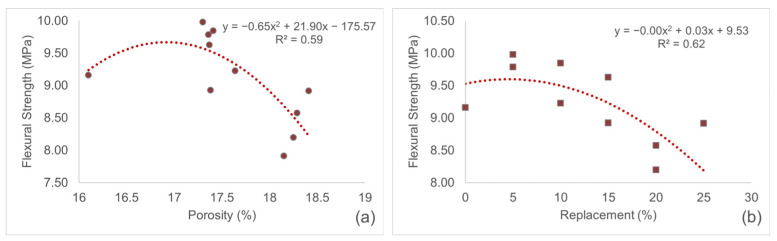
(**a**) Relationship between flexural strength and porosity of mortars. (**b**) Relationship between flexural strength and replacement ratio of mortars.

Examination of Figure 13 indicates a moderate correlation between both porosity and flexural strength, as well as replacement ratio and flexural strength. Second-order polynomial regressions were applied to both relationships, with R^2^ values of 0.59 and 0.62 for flexural strength versus porosity and replacement ratio, respectively, indicating moderate correlations. These findings suggest that while porosity and replacement ratio influence strength to a certain extent, the variation in strength is largely governed by other factors. The incorporation of inert mineral powders can limit cement hydration, thereby reducing the formation of binding phases. Although porosity exhibits certain trends, the particle characteristics, binding capacity, and microstructural interactions of the replacement materials play a more dominant role in determining flexural strength. Furthermore, the filler effect of the mineral powders and their interaction with the matrix contribute to the nonlinearity of the relationship between replacement ratio and strength.

### 3.5. Compressive Strength Test Results

**Figure 14:**
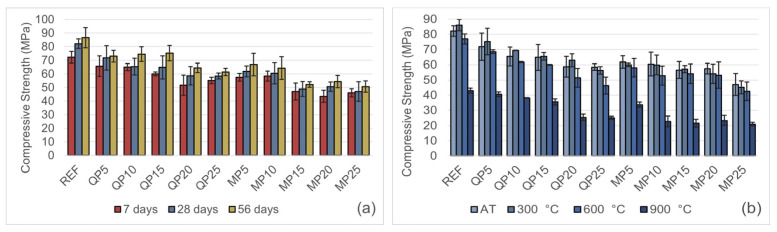
(**a**) Change in compressive strength at 7, 28, and 56 days of curing. (**b**) Compressive strength test results after exposure to AT, 300, 600, and 900 °C.

The average compressive strengths of the mortar specimens at 7, 28, and 56 days are presented in Figure 14a. In all mortar mixes, compressive strength generally increased with curing time, indicating the continued hydration of cement and the progressive development of strength. The highest compressive strength was observed in the control specimen. As the replacement ratio increased, the compressive strength generally decreased in both the QP and MP groups. However, at each replacement level, the MP specimens exhibited lower strength compared to the corresponding QP specimens, indicating a more favorable performance of the QP group.

The effect of elevated temperature on compressive strength is shown in Figure 14b. In all mixes, increasing the replacement ratio led to a significant decrease in compressive strength at elevated temperatures. Nevertheless, a temporary increase in compressive strength was observed at 300 °C, similar to the trend seen in flexural strength for REF, QP5, QP10, QP15, and QP20 specimens.

In contrast, the compressive strength of the MP specimens continuously decreased with increasing temperature. This behavior is associated with the loss of stability of the mineral structure of cultured marble at high temperatures and the decomposition of CaCO_3_, which accelerates the formation of microcracks and weakens the matrix integrity, as reported in the literature [27]. Additionally, at all temperatures, WQP-containing specimens exhibited higher compressive strength than WMP-containing specimens, indicating that WQP provides a more effective filling and binding enhancement within the mortar matrix.

**Figure 15:**
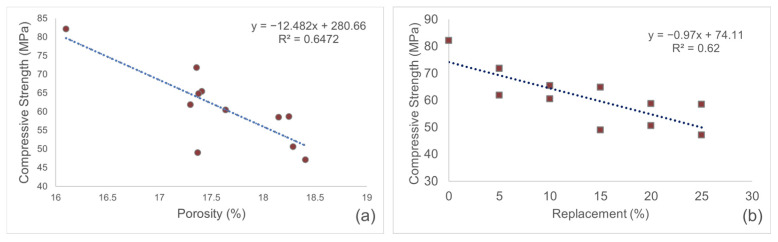
(**a**) Relationship between compressive strength and porosity of mortars. (**b**) Relationship between compressive strength and replacement ratio of mortars.

Examination of Figure 15 indicates a moderate-to-strong correlation between compressive strength and porosity, with an $R^{2}=0.6472$, and a similar effect observed for the relationship between replacement ratio and compressive strength, with an $R^{2}=0.62$. Although the incorporated WP limits cement hydration, variations in porosity and replacement ratio partially account for the observed changes in compressive strength. Furthermore, particle size, filler effect, and microstructural interactions of the mineral powders emerge as additional critical factors influencing strength.

### 3.6. Abrasion Resistance Test Results

**Figure 16:**
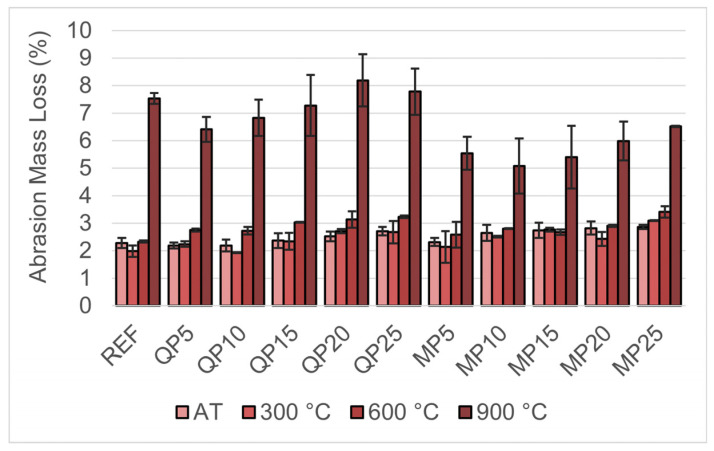
Abrasion mass loss results after exposure to AT, 300, 600, and 900 °C.

The abrasion mass loss results after exposure to AT, 300, 600, and 900 °C are presented in Figure 16. In general, abrasion tests conducted under AT conditions showed that mass loss increased with higher replacement ratios in both WQP- and WMP-containing groups. At 300 °C, some specimens exhibited a slight decrease in mass loss, indicating improved wear resistance at this temperature.

At 600 °C and 900 °C, specimens containing WMP exhibited lower wear losses compared to those incorporating WQP. The higher wear rate of WQP at these temperatures may be related to microstructural changes in quartz grains, as reported in previous studies [28]. Exposure to high temperatures compromised the integrity of the mortar matrix and increased porosity, contributing to the observed increase in abrasion losses. At 900 °C, a sharp increase in wear losses was observed in all specimens.

The general trends of the experimental results obtained in this study relative to the reference mixture are summarized in Table 5.

### 3.7. Microstructure Analysis Results

The SEM analysis results of REF specimens subjected to different temperatures are presented below.

**Figure 17:**
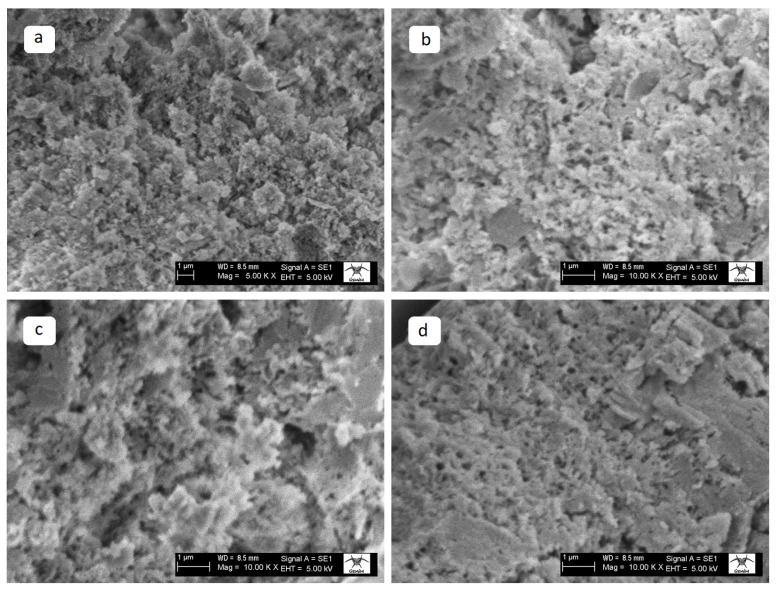
SEM images of the REF specimens at (**a**) AT, (**b**) 300 °C, (**c**) 600 °C, and (**d**) 900 °C.

The SEM images of REF specimens exposed to different temperatures are presented in Figure 17. At ambient temperature, the specimen exhibited a compact microstructure with limited porosity and a homogeneous distribution of C–S–H-based hydration products. The aggregate–paste interface remained intact, and a typical self-compacted mortar formation was observed. At 300 °C, the matrix showed signs of degradation; the loss of hydration water led to the formation of fine microcracks and superficial disintegration, while partial decomposition of the C–S–H phase was also observed. Upon exposure to 600 °C, the microcracks widened and significant decomposition of the C–S–H phase occurred, resulting in a looser and more granular binder paste. At 900 °C, the matrix was severely decomposed, porosity increased, and the microstructure lost its ductility, exhibiting a glassy and brittle appearance.

**Figure 18:**
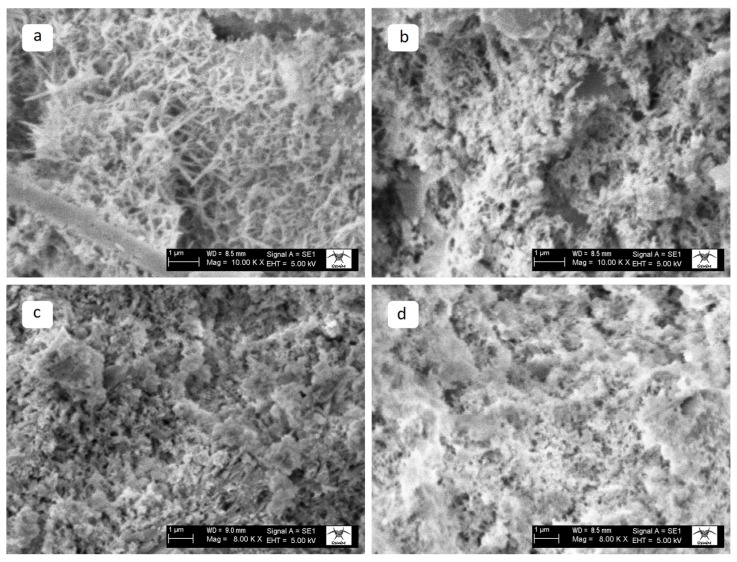
SEM images of the QP25 specimens at (**a**) AT, (**b**) 300 °C, (**c**) 600 °C, and (**d**) 900 °C.

The SEM images of the QP25 specimens exposed to different temperatures are presented in Figure 18. At ambient temperature, the WQP-containing specimen exhibits a compact and interconnected C–S–H network with dense needle-like ettringite crystals, and the fine-grained mineral additive contributes to reduced porosity. At 300 °C, matrix degradation is observed; however, the matrix integrity is comparatively better preserved than in the control specimen, indicating that the void-filling effect of WQP enhances structural stability. At 600 °C, matrix degradation remains limited. Although dehydration-induced cracks are present, the matrix is still partially preserved, with WQP contributing to maintaining structural stability. At 900 °C, matrix degradation becomes more pronounced, but porosity and overall matrix decomposition are less severe than in the control specimen. Overall, WQP incorporation exerts a partial yet significant positive effect on high-temperature performance.

The SEM analysis results of WMP-containing specimens at different temperatures are presented below.

**Figure 19:**
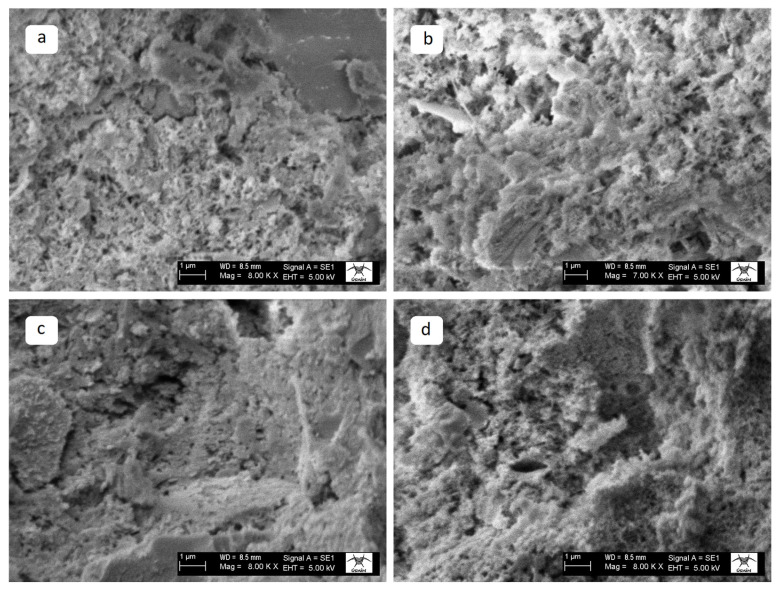
SEM images of the MP25 specimens at (**a**) AT, (**b**) 300 °C, (**c**) 600 °C, and (**d**) 900 °C.

The SEM images of the MP25 specimens exposed to different temperatures are presented in Figure 19. At ambient temperature, the WMP-containing specimen exhibits a compact structure; however, its matrix morphology is more irregular and heterogeneous compared to the WQP-substituted specimen. The filler effect contributes to a reduction in porosity. At 300 °C, matrix degradation is more pronounced than in the WQP-substituted specimen, indicating that the mineralogical composition of WMP limits its thermal stability. At 600 °C, matrix degradation accelerates, the matrix becomes looser, and the pore connectivity increases. Although WMP provides limited improvement compared to the control specimen, it remains less effective than the WQP-substituted specimen. At 900 °C, severe matrix degradation and high porosity are observed. Overall, WMP incorporation has a limited effect on high-temperature performance and does not preserve matrix integrity as effectively as WQP-substituted specimens.

**Figure 20:**
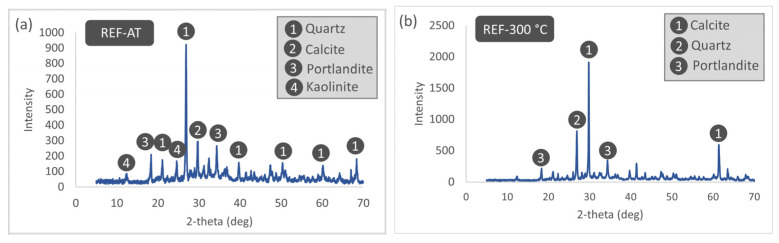

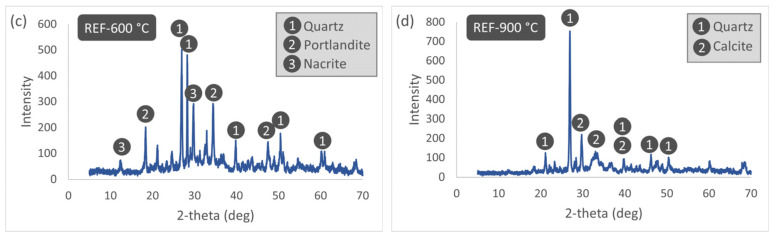
XRD patterns of REF specimens subjected to different thermal treatment temperatures. (**a**) AT, (**b**) 300 °C, (**c**) 600 °C, (**d**) 900 °C.

The XRD patterns of the REF specimens subjected to different thermal treatment temperatures are presented in Figure 20. XRD analyses of the REF specimens under AT conditions show distinct diffraction peaks corresponding to quartz, calcite, portlandite, and kaolinite. When the temperature is increased to 300 °C, the kaolinite peak disappears, whereas a pronounced increase in the intensity of the calcite peak is observed. This increase may be attributed to enhanced crystallinity resulting from extensive dehydration within the mortar matrix or to the acceleration of carbonation reactions under elevated temperature. At 600 °C, the calcite phase within the cement matrix undergoes substantial degradation or becomes completely depleted due to progressive thermal decomposition; at the same temperature, the portlandite peak intensity also decreases markedly and tends toward disappearance. Within this temperature range, the high-temperature polymorph of the clay mineral, nacrite, becomes evident, while the quartz phase retains its structural stability. Finally, at 900 °C, the complete decomposition of portlandite and other hydrated phases results in a mortar matrix composed predominantly of the thermally more stable quartz and calcite phases. The presence of calcite at this temperature—despite its expected calcination—can be explained by the recarbonation of calcium oxide formed during heating through reaction with atmospheric carbon dioxide during the cooling stage.

**Figure 21:**
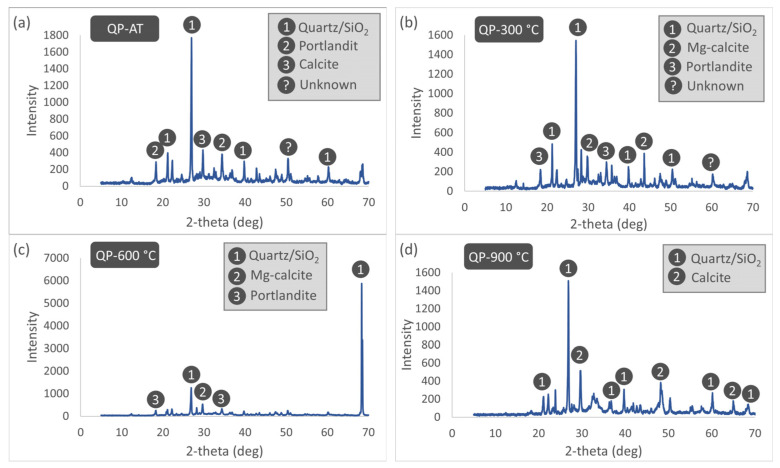
XRD patterns of QP25 specimens subjected to different thermal treatment temperatures. (**a**) AT, (**b**) 300 °C, (**c**) 600 °C, (**d**) 900 °C.

The XRD patterns of the QP25 specimens subjected to different thermal treatment temperatures are presented in Figure 21. XRD analyses of the 25% WQP-substituted specimens under AT conditions reveal the presence of quartz derived from sand and the replacement material, alongside portlandite as a cement hydration product and carbonate-based calcite. When the temperature is increased to 300 °C, extensive dehydration of the mortar begins, resulting in the disappearance of the calcite phase from the diffraction pattern, while portlandite remains present. Additionally, Mg-calcite phases, which are not observed under AT conditions, are detected at this temperature. Upon reaching 600 °C, a pronounced increase in the intensity of the quartz peaks is observed compared to lower temperatures. This significant increase can be attributed to the accelerated calcination of thermally unstable hydrated phases and carbonates, which renders the relatively thermally stable quartz phase dominant in the X-ray diffraction pattern. At this temperature, pure calcite is no longer observed, whereas portlandite and Mg-calcite phases are still detected. At 900 °C, thermal decomposition and phase transformations are largely complete, and the matrix is dominated by quartz and the reformed calcite phase. The reappearance of calcite at this temperature is explained by the recarbonation of calcium oxide, formed at high temperatures, through reaction with atmospheric carbon dioxide during cooling. Overall, WQP substitution enhances the thermal stability of the quartz phase, while the decomposition and reformation behavior of the calcite and Mg-calcite phases exhibits complex temperature-dependent variations.

**Figure 22:**
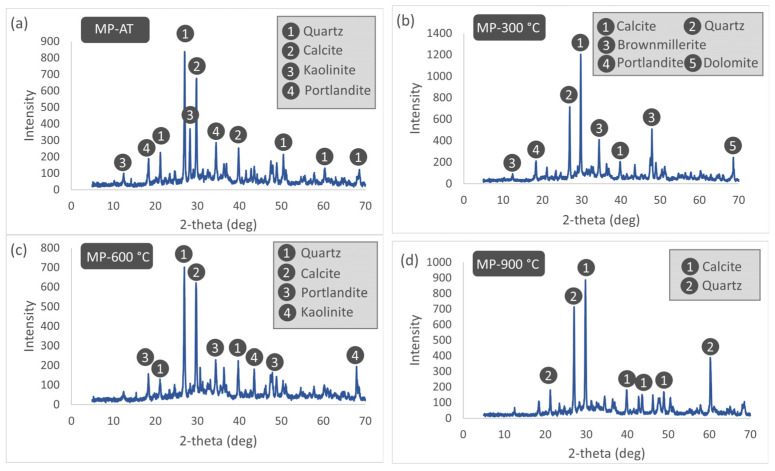
XRD patterns of MP25 specimens subjected to different thermal treatment temperatures. (**a**) AT, (**b**) 300 °C, (**c**) 600 °C, (**d**) 900 °C.

The XRD patterns of the MP25 specimens subjected to different thermal treatment temperatures are presented in Figure 22. The XRD analysis of the 25% WMP-substituted mortar at room temperature revealed the presence of quartz originating from the aggregates, portlandite as a cement hydration product, and calcite and kaolinite phases contributed by the WMP substitution and cement additives. Upon heating to 300 °C, intense dehydration of the mortar was observed, leading to the disappearance of the kaolinite phase, while brownmillerite and dolomite phases became pronounced due to high-temperature effects. At 600 °C, although the decomposition of thermally unstable hydrated phases had begun, portlandite, calcite, and kaolinite were still detectable, suggesting that the matrix containing WMP partially delayed the thermal phase transformations. At 900 °C, despite the substantial completion of thermal decomposition, the mortar matrix was dominated by thermally stable aggregate-derived quartz and calcite phases exhibiting high peak intensities. The persistence of calcite as a dominant phase at this elevated temperature may be related to the partial suppression of calcination caused by the release of local CO_2_ from organic components within WMP and to the re-carbonation of CaO formed during high-temperature exposure upon cooling. Moreover, the decomposition of other thermally unstable phases at elevated temperatures contributed to the relative dominance of calcite in the XRD patterns.

In general, the use of WQP and WMP influences the microstructure of self-compacting mortars through particle characteristics and filler effects. SEM analyses show that mortars containing WQP exhibit a denser and more homogeneous matrix with fewer microcracks and lower pore connectivity, whereas WMP-containing mortars, particularly at higher replacement levels, display more microcracks and larger pores. XRD results further confirm that the high-temperature behavior is largely determined by the thermal stability of the dominant mineral phases: WQP forms a quartz-rich framework that remains stable at elevated temperatures, while WMP preserves partial matrix integrity due to the slower decomposition of calcite. These observations explain the differences in mechanical performance and durability between the two types of mortars.

## 4. Conclusions

This study evaluates the potential use of waste powders derived from cultured marble and quartz-based composite plates as partial cement replacements in self-compacting mortars. The effects of these powders on fresh properties, mechanical performance, durability, and microstructural characteristics were systematically investigated, providing mechanistic insights into how filler type and replacement level influence mortar behavior. The findings offer practical guidelines for sustainable SCM production and highlight the contribution of industrial waste materials to environmental and construction sustainability.

The increase in the replacement ratio generally led to higher water demand and reduced workability. Mortars containing WQP exhibited a higher unit weight compared to those containing WMP. This is thought to result from the particle morphology, as observed in SEM images, with WMP particles being more rounded and WQP particles being angular and irregular. The filler efficiency and particle distribution provided by the angular morphology of WQP contributed to a denser and more homogeneous mortar matrix. Porosity remained slightly above the control at low to medium replacement levels (up to ~15%) but increased more noticeably at higher replacement levels. Sorptivity values were higher than the control for all replacement ratios, without a consistent trend with increasing replacement.

Flexural strength showed a slight improvement at low replacement levels due to the filler effect, with performance comparable to the control maintained up to 10% for both WQP and WMP. Beyond this replacement ratio, flexural strength generally decreased. Compressive strength decreased with increasing replacement levels; however, mixtures containing WQP consistently exhibited higher compressive strength than those containing WMP, indicating a more effective filler contribution of WQP. Although compressive strength values remained lower than the control mixture, replacement levels up to 10% for both WQP and WMP maintained acceptable and consistent strength across curing ages and exposure temperatures. Abrasion resistance generally declined with increasing replacement ratios; nevertheless, mixtures with low replacement levels, particularly up to 10% for both WQP and WMP, exhibited abrasion mass losses comparable to or lower than the control, which can be attributed to the micro-filler effect.

SEM analyses revealed that WQP-containing mortars had a more tightly packed and homogeneous microstructure with fewer microcracks and reduced pore connectivity, whereas WMP-containing mortars exhibited higher microcrack density and larger pores, particularly at higher replacement levels. XRD results showed that the high-temperature performance of the mortars was largely determined by the thermal stability of the dominant mineral phases: WQP contributed to a quartz-rich framework that remained stable at elevated temperatures, while WMP maintained partial matrix integrity due to the slower decomposition of calcite, which helped to limit matrix deterioration.

At low replacement levels, the use of WQP and WMP exhibited acceptable performance in terms of compressive strength, flexural strength, porosity, water absorption, and abrasion resistance. These mixtures, at replacement levels up to 10% for both WQP and WMP, are suitable for non-structural applications or areas exposed to normal environmental conditions, providing a practical guideline for construction use. Utilizing these industrial waste powders as replacement materials can contribute to minimizing the environmental impact of cement. Furthermore, incorporating these wastes into mortar production provides a sustainable solution for waste management and helps reduce environmental pollution. It also provides significant potential for producing eco-friendly construction materials.

## Figures and Tables

**Table 1:** Literature comparison of natural and engineered stone powders as cement replacements in SCM/SCC.

Reference	Waste Type	Material Nature	Cement Replacement (%)	Matrix Type	Elevated Temperature	Microstructural Analysis
Hameed et al. [10]	Marble powder	Natural stone	5–10–15–20	Self-compacting concrete	No	No
Karakurt & Dumangöz [11]	Marble powder, BFS	Natural stone, slag	10–20–30	Self-compacting concrete	No	No
Prakash et al. [12]	Marble powder	Natural stone	5–10–20–30–40–50	Cementitious composites	No	SEM
Abouelnour et al. [13]	Marble and granite powders	Natural stone	2–4–6–8–10	Cementitious composites	No	SEM
Sevinç & Durgun [14]	Basalt, pumice and barite powders	Natural stone	7.5 and 15	Mortar	Up to 600 °C	SEM, XRD, TGA
Abuqasim et al. [16]	Porcelain polishing residue (PPR)	Engineered stone	5–10–20	Mortar	No	SEM, EDS
Present study	WQP & WMP (Artificial plate powders)	Engineered stone (mineral + resin)	5–10–15–20–25	Self-compacting mortar	300, 600, 900 °C	SEM, XRD

Note: For some studies, microstructural analyses were limited to mechanical or macro-level evaluations without detailed SEM/XRD under elevated temperatures, thus summarized conservatively.

**Table 2:** Chemical compositions, physical and mechanical properties of Portland cement.

	Properties	Value
	SiO_2_	18.76
	Al_2_O_3_	4.76
	Fe_2_O_3_	3.13
	CaO	63.48
	MgO	1.02
Chemical Compositions (%)	SO_3_	3.08
	Loss on ignition	3.87
	Na_2_O	0.16
	K_2_O	0.97
	Na_2_O + 0.658 K_2_O	0.80
	S.CaO	1.86
	Specific gravity, g/cm^3^	3.15
	Initial setting time, minute	155
Physical Properties	Final setting time, minute	195
	Soundness (Le Chatelier), mm	2.0
	Specific surface, cm^2^/g	3940
Mechanical Properties (MPa)	Compressive strength (1 day)	17.7
	Compressive strength (2 days)	28.1
	Compressive strength (7 days)	38.4
	Compressive strength (28 days)	54.5

**Table 3:** Chemical compositions of WQP and WMP.

Analysis Report	WQP (%)	WMP (%)
SiO_2_	84.126	1.125
Al_2_O_3_	0.767	0.123
Fe_2_O_3_	0.964	0.081
CaO	0.701	44.451
MgO	0.055	1.533
P_2_O_5_	0.020	0.015
Na_2_O	0.311	0.027
K_2_O	0.076	0.021
TiO_2_	0.773	0.012
SrO	0.014	0.014
PbO	0.001	0.003
Loss on ignition	12.220	52.47

**Table 4:** Mix quantities of cement mortars containing WMP and WQP.

NO	Mix ID	Cement (g)	Sand (g)	WMP (g)	WQP (g)	Water (g)	HRWR (g)
1	REF	600	1529	0	-	222	8
2	MP5	570	1529	30	-	222	8
3	MP10	540	1529	60	-	222	9
4	MP15	510	1529	90	-	222	9
5	MP20	480	1529	120	-	222	9.5
6	MP25	450	1529	150	-	222	9.5
7	QP5	570	1529	-	30	222	8
8	QP10	540	1529	-	60	222	9
9	QP15	510	1529	-	90	222	10
10	QP20	480	1529	-	120	222	10
11	QP25	450	1529	-	150	222	10

**Table 5:** Summary of performance trends in mortars containing WQP and WMP.

Mix ID	Workability	Porosity	Bulk Specific Gravity	Sorptivity	Flexural Strength			
					AT	300 °C	600 °C	900 °C
REF	Ref.	Ref.	Ref.	Ref.	Ref.	Ref.	Ref.	Ref.
QP5	↓	↑	↓	↑↑↑	↑↑	↑↑	↑↑	↑↑
QP10	↓↓↓	↑	↓↓	↑↑↑	↑↑	↓	↑↑	↑↑
QP15	↓↓↓	↑	↓	↑↑↑	↓	↓	≈	≈
QP20	↓↓↓	↑	↓↓	↑↑↑	↓↓	↓↓	↓↓	↓↓
QP25	↓↓↓	↑	↓↓	↑↑↑	↓↓	↓↓	↓↓	↓↓
MP5	≈	↑	↓↓	↑↑↑	↑↑	↓	≈	↑↑
MP10	↓	↑	↓↓	↑↑↑	≈	↓	↓	↑↑
MP15	↓↓↓	↑	↓↓↓	↑↑↑	↑	↓↓	↓	↑
MP20	↓↓↓	↑	↓↓↓	↑↑↑	↓↓	↓↓↓	↓↓	↓
MP25	↓↓↓	↑	↓↓↓	↑↑↑	↓↓	↓↓↓	↓↓	↓↓
**Mix ID**	**Compressive Strength**				**Abrasion Mass Loss**			
	**AT**	**300 °C**	**600 °C**	**900 °C**	**AT**	**300 °C**	**600 °C**	**900 °C**
REF	Ref.	Ref.	Ref.	Ref.	Ref.	Ref.	Ref.	Ref.
QP5	↓	↓	↓	≈	↓↓	↑	↑↑	↓↓
QP10	↓↓	↓↓	↓↓	↓	↓↓	≈	↑↑	↓↓
QP15	↓↓	↓↓↓	↓↓↓	↓↓	↑	↑	↑↑↑	↓
QP20	↓↓↓	↓↓↓	↓↓↓	↓↓↓	↑↑	↑↑↑	↑↑↑	↑↑
QP25	↓↓↓	↓↓↓	↓↓↓	↓↓↓	↑↑↑	↑↑↑	↑↑↑	↑
MP5	↓↓	↓↓↓	↓↓	↓↓	↑	↑	↑	↓↓↓
MP10	↓↓	↓↓↓	↓↓↓	↓↓↓	↑↑	↑↑↑	↑↑↑	↓↓↓
MP15	↓↓↓	↓↓↓	↓↓↓	↓↓↓	↑↑	↑↑↑	↑↑	↓↓↓
MP20	↓↓↓	↓↓↓	↓↓↓	↓↓↓	↑↑↑	↑↑↑	↑↑↑	↓↓↓
MP25	↓↓↓	↓↓↓	↓↓↓	↓↓↓	↑↑↑	↑↑↑	↑↑↑	↓↓

Note: Arrows indicate the trend relative to the control mixture. ↑ and ↓ represent an increase and decrease, respectively. The number of arrows denotes the relative magnitude of change (↑/↓: slight, ↑↑/↓↓: moderate, ↑↑↑/↓↓↓: significant). ≈ indicates no notable change.

## Data Availability

The original contributions presented in this study are included in the article. Further inquiries can be directed to the corresponding author.

## Abbreviations

The following abbreviations are used in this manuscript:

WP	Waste powder
WQP	Quartz-based plate powder
WMP	Cultured marble powder
REF	Control specimens without waste powder
QP	Specimens containing waste quartz-based plate powder
MP	Specimens containing waste marble powder
SCM	Self-compacting mortar
HRWR	High-range water reducer

## References

[1] Khaiyum M.Z., Sarker S., Kabir G. (2023). Evaluation of carbon emission factors in the cement industry: An emerging economy context. Sustainability.

[2] Raffetti E., Treccani M., Donato F. (2019). Cement plant emissions and health effects in the general population: A systematic review. Chemosphere.

[3] Mohamad N., Muthusamy K., Embong R., Kusbiantoro A., Hashim M.H. (2022). Environmental impact of cement production and solutions: A review. Mater. Today Proc..

[4] Scrivener K.L., John V.M., Gartner E.M. (2018). Eco-efficient cements: Potential economically viable solutions for a low-CO_2_ cement-based materials industry. Cem. Concr. Res..

[5] Yang L., Hu X., Liu Y., Zhou D., Yuan B., Liu S., Luo Z., Li X., Jin D., Xu F. (2026). Multiscale characterization of geopolymers modified with alkali-catalyzed nano-silica: Effects on dispersion and mechanical properties. Cem. Concr. Compos..

[6] Aydın G., Karakurt İ. (2020). Evaluation of natural stone production and processing plant wastes. ALKU J. Sci..

[7] Sagar G.S., Mukthi S., Mehta V. (2025). Analyzing compressive, flexural, and tensile strength of concrete incorporating used foundry sand: Experimental and machine learning insights. Arch. Comput. Methods Eng..

[8] İrmak Er A., Yazıcıoğlu S. (2025). Investigation of mechanical properties of granite waste sludge and sepiolite substituted self-compacting mortars. Gazi Univ. J. Sci..

[9] Benjeddou O., Alwetaishi M. (2021). Valorization of powder obtained from marble sludge waste and its suitability as a mineral filler. Crystals.

[10] Hameed A., Qazi A.U., Abbas S., Rehman A. (2016). Self compacting concrete: Use of waste marble powder as filler material. Pak. J. Eng. Appl. Sci..

[11] Karakurt C., Dumangöz M. (2022). Rheological and durability properties of self-compacting concrete produced using marble dust and blast furnace slag. Materials.

[12] Prakash B., Saravanan T.J., Kabeer K.I.S.A., Bisht K. (2023). Exploring the potential of waste marble powder as a sustainable substitute to cement in cement-based composites: A review. Constr. Build. Mater..

[13] Abouelnour M.A., EL-Aziz M.A.A., Osman K.M., Fathy I.N., Tayeh B.A., Elfakharany M.E. (2024). Recycling of marble and granite waste in concrete by incorporating nano alumina. Constr. Build. Mater..

[14] Sevinç A.H., Durgun M.Y. (2023). Elevated temperature performance of cementitious mortars containing pumice, barite, and basalt powder. J. Build. Eng..

[15] Huseien G.F., Sam A.R.M., Faridmehr I., Hajmohammadian Baghban M. (2021). Performance of epoxy resin polymer as self-healing cementitious materials agent in mortar. Materials.

[16] Abuqasim S., Ersoy S., Kurtay-Yildiz M., Öztürk İ.Ş., Sari F.A., Emiroğlu M. (2025). Examination of porcelain polishing residue as a supplementary cementitious materials. J. Build. Eng..

[17] Bayraktar O.Y., Tunçtan M., Benli A., Türkel İ., Kızılay G., Kaplan G. (2024). A Study on sustainable foam concrete with waste polyester and ceramic powder: Properties and durability. J. Build. Eng..

[18] Evren Ö. (2018). Manufacture and Characterization of Glass Fiber Reinforced Artificial Marble Blocks from Marble Wastes. Master’s Thesis.

[19] (2011). Admixtures for Concrete, Mortar and Grout–Part 2: Concrete Admixtures–Definitions, Requirements, Conformity, Marking and Labelling.

[20] Sika (2025). Sika^®^ ViscoCrete^®^ ACE 450—High Range Water Reducing, Superplasticizer for Concrete. Technical Data Sheet. Sika Türkiye. https://tur.sika.com/dms/getdocument.get/fa5e6b94-94d6-4800-b614-2289dbca5ccd/sika-viscocrete-ace450.pdf.

[21] (2015). Fire-Resistance Tests—Elements of Building Construction.

[22] (2003). Standard Test Methods for Fire Tests of Building Construction and Materials.

[23] (2009). Methods of Testing Cement—Part 1: Determination of Strength.

[24] Akgül M., Etli S. (2024). Investigation of the variation of mechanical and durability properties of elements manufactured with rubber substituted SCMs with element height. Constr. Build. Mater..

[25] (2013). Standard Test Method for Measurement of Rate of Absorption of Water by Hydraulic Cement Concretes.

[26] Borucka-Lipska J., Brzozowski P., Błyszko J., Bednarek R., Horszczaruk E. (2020). Effects of elevated temperatures on the properties of cement mortars with the iron oxides concentrate. Materials.

[27] Petkova V., Stoyanov V., Kostova B., Kostov-Kytin V., Kalinkin A., Zvereva I., Tzvetanova Y. (2021). Crystal-chemical and thermal properties of decorative cement composites. Materials.

[28] Dudek M., Sitarz M. (2020). Analysis of changes in the microstructure of geopolymer mortar after exposure to high temperatures. Materials.

